# Editorial: Unveiling functional and structural physiological transformations through muscle stretching

**DOI:** 10.3389/fphys.2025.1668127

**Published:** 2025-07-30

**Authors:** Emiliano Cè, Stefano Longo, Jacob Caldwell

**Affiliations:** ^1^ Department of Biomedical Sciences for Health, Universitá degli Studi di Milano, Milan, Italy; ^2^ Integrative Human Performance Laboratory, Department of Exercise and Sport Science, University of Wisconsin-La Crosse, La Crosse, WI, United States

**Keywords:** acute and chronic stretching, autonomic, neuromuscular, cardiovascular, hemodynamics, low intensity exercise, microvascular

Muscle stretching is widely recognized as a fundamental component of physicl activity and exercise routines, traditionally applied to improve joint flexibility and increase range of motion (ROM). However, a growing body of evidence suggests that stretching may elicit a broader spectrum of physiological effects beyond musculoskeletal adaptations. Emerging studies demonstrate that stretching can influence neuromuscular performance, cardiovascular function, autonomic nervous system activity, and tissue-level mechanical properties. These effects, which can occur both acutely and chronically, open new perspectives on stretching as a low-intensity yet impactful form of physical intervention suitable for diverse populations, including clinical and aging cohorts.

The Research Topic *Unveiling Functional and Structural Physiological Transformations Through Muscle Stretching*—hosted in *Frontiers in Physiology*—was conceived to bring together original contributions and comprehensive reviews that explore the structural and functional adaptations induced by stretching. Our aim was to provide an integrative view of how stretching, through its various modalities and protocols, can influence physiological systems in health and disease. The papers included in this Research Topic explore these dimensions across methodological, mechanistic, and application-oriented perspectives.

The first study, conducted by Nakamura et al., focused on older adults, a population in which flexibility loss and musculoskeletal stiffness are common and often associated with impaired mobility and increased fall risk. The authors investigated the effects of a 10-week static stretching (SS) intervention performed at least three times per week targeting the ankle plantar flexor muscles. The intervention led to a significant increase in dorsiflexion ROM and a moderate reduction in medial gastrocnemius tissue hardness. However, no correlation was observed between these two outcomes, suggesting that the improvements in ROM were more likely mediated by enhanced stretch tolerance rather than by a direct reduction in muscle stiffness. These findings reinforce the idea that perceptual and neural factors may contribute significantly to stretching-induced flexibility gains, particularly in older individuals.

The second article, by Kranjc et al., examined the acute mechanical effects of proprioceptive neuromuscular facilitation (PNF) stretching on *rectus femoris* muscle stiffness in healthy young adults. Despite the well-established use of PNF stretching in clinical and athletic settings, the authors found no statistically significant changes in shear modulus following different volumes of PNF stretching compared to passive rest. Interestingly, they observed a consistent difference in muscle stiffness between proximal and distal measurement sites, independent of the intervention. This site-specific disparity underscores the need for anatomical precision in elastography-based assessments and suggests that local muscle architecture may influence the apparent mechanical response to stretching. These results challenge the assumption that PNF stretching necessarily leads to immediate reductions in muscle stiffness and highlight the importance of further investigating the specific neuromechanical pathways involved.

In a more applied context, Wang et al. investigated the acute effects of both static and dynamic stretching on neuromuscular performance and balance in recreationally active young men. Participants were allocated to three groups—static stretching, dynamic stretching, or no stretching—and completed pre- and post-intervention assessments, including various balance, change-of-direction, and jump performance tests. The results demonstrated that while static stretching improved static balance, dynamic stretching had broader benefits, enhancing performance in multiple tests of dynamic balance, coordination, and power output. These findings support the specificity of stretching-induced neuromuscular adaptations and suggest that dynamic stretching may be more suitable when immediate improvements in performance are desired, such as in pre-competition warm-ups or functional rehabilitation programs.

In a fourth contribution, Nakamura et al. explored how to sustain the effects of static stretching beyond the typical acute timeframe. They investigated whether a brief (15-second) additional stretch applied during a rest period could prolong the benefits of an initial 180-second SS session. Measures of knee flexion ROM, tissue hardness, and pressure pain threshold were collected at multiple time points. Their findings revealed that the short supplementary stretch effectively extended the improvements in ROM and reduced tissue hardness for up to 30 min after the intervention. This study provides a practical and innovative insight into how small protocol adjustments can optimize the time course of stretching effects, potentially offering enhanced strategies for use in both therapeutic and athletic settings.

Finally, Vicari et al. investigated the relevance of muscle flexibility in non-traditional application: saddle pressure distribution in young competitive cyclists. Using the V sit-and-reach test to evaluate hamstring and lower back flexibility and measuring anterior saddle pressure during cycling at varying intensities, the authors found that flexibility was a significant predictor of pressure values in the anterior saddle region. Specifically, greater flexibility was associated with reduced anterior pressure, which may be beneficial for comfort, posture, and injury prevention during prolonged cycling. These findings introduce a new angle on the functional importance of stretching and suggest potential applications in equipment ergonomics and youth sports development.

Taken together, these five studies emphasize the complexity of the physiological responses to stretching. They highlight the need for more refined experimental designs that account for factors such as stretching modality (static, dynamic, or PNF), duration, intensity, anatomical target, and the timing of assessments. Moreover, the divergent outcomes reported across different populations—ranging from adolescents to older adults—point to the importance of individual characteristics, including age, training status, and flexibility baseline, in modulating stretching effects.

Importantly, this Research Topic contributes to the growing consensus that stretching is not a passive or simplistic practice. Rather, it should be viewed as a structured, adaptable, and evidence-based form of exercise that holds potential for systemic physiological modulation. The data provided by these studies pave the way for further research aimed at elucidating the underlying mechanisms—whether neural, mechanical, or hemodynamic—that drive the observed changes. There is also a pressing need to explore how stretching interventions interact with other forms of physical activity and how they can be integrated into personalized exercise prescriptions for health promotion and disease prevention.

In conclusion, we believe this Research Topic offers an important step toward repositioning stretching as a scientifically grounded intervention capable of eliciting meaningful adaptations in human physiology. We hope that the insights presented here will stimulate further multidisciplinary collaboration and encourage more nuanced, mechanistic research in this area. Whether used to improve mobility in aging populations, to support recovery and performance in athletes, or to optimize ergonomics in sport-specific contexts, stretching deserves continued attention as a low-intensity, accessible, and potentially powerful therapeutic tool.

